# Resident and Fellow Perspectives on Family Planning and Building During Training

**DOI:** 10.1001/jamanetworkopen.2024.29345

**Published:** 2024-08-28

**Authors:** E. Shirin Dason, Abby Kapsack, Nancy N. Baxter, Dionne Gesink, Heather Shapiro, Andrea N. Simpson

**Affiliations:** 1 University of Toronto Temerty Faculty of Medicine, Department of Obstetrics and Gynecology, Division of Reproductive Endocrinology and Infertility, Mount Sinai Hospital, Toronto, Canada; 2 University of Toronto Temerty Faculty of Medicine, Toronto, Canada; 3 University of Toronto Temerty Faculty of Medicine, Department of Surgery, Li Ka Shing Knowledge Institute, St Michael’s Hospital Unity Health Toronto, Toronto, Canada; 4 Faculty of Medicine and Health, University of Sydney, Sydney, New South Wales, Australia; 5 University of Toronto Dalla Lana School of Public Health, Toronto, Canada

## Abstract

**Importance:**

Physicians, especially surgeons, delay family building, and face age-related complications of pregnancy.

**Objective:**

To explore factors that are associated with family building decisions among residents and fellows and to understand their thoughts on accessing family building supports, workplace culture, and attitudes toward family planning.

**Design, Setting, and Participants:**

This qualitative study was conducted from May to August 2022 at postgraduate training sites across Ontario, Canada. Participants included 29 postgraduate learners. Purposive sampling from 80 eligible trainees was used to ensure diverse demographics, including sex, specialty, and institution. Semi-structured individual interviews lasting 30 to 90 minutes were conducted over a virtual platform. Participants were asked to share their thoughts and experiences on family planning in a medical career, family building goals, the role of mentorship, and knowledge of current family planning supports.

**Main Outcomes and Measures:**

Themes associated with residents’ perception of family planning within the context of a medical career.

**Results:**

Twenty-nine trainees, ages 25 to 37 years old (22 [75.9%] were female, 26 [89.7%] were heterosexual; median [range] age, 30 [25-37] years) from various institutions took part in this study. The participants included both 24 residents (82.8%) and 5 fellows (17.2%) and 8 (27.6%) were from surgical specialties, 3 (10.3%) from pediatrics and subspecialties, 4 (13.8%) from internal medicine and subspecialties, 2 (6.9%) from obstetrics and gynecology, 3 (10.3%) family medicine, 2 (6.9%) from anesthesia, and 7 (24.1%) other medical specialties. Four themes were identified: (1) tension between role as a physician and role as a parent; (2) impact of role models and mentorship on family planning choices; (3) family building is discouraged during training, especially in surgical specialties; and (4) need for tangible family planning supports in training.

**Conclusions and Relevance:**

In this qualitative study of residents’ and fellows’ perspectives on family building, trainees did not perceive residency and fellowship programs as supportive for individuals who want to family build during training leading to personal and professional dissonance. Trainees identified that more tangible supports, transparent information, and mentorship may improve a trainees’ feeling of support.

## Key Points

**Question:**

What factors do medical trainees consider when making family planning or building decisions?

**Findings:**

This qualitative study of 29 residents identified 4 themes: (1) tension between role as a physician and role as a parent; (2) impact of role models and mentorship on family planning choices; (3) family building is discouraged during training, especially in surgical specialties; and (4) need for tangible family planning supports in training.

**Meaning:**

These findings suggest that trainees perceived the current model of medical training as unsupportive of family building or parenting, and this affects their family planning.

## Teaser

This qualitative study examines factors associated with family planning decisions among residents and fellows at training sites in Canada.

## Introduction

Physicians often delay childbearing until the completion of postgraduate training.^1^ Resident physicians are less likely than nonphysicians to have a child during their peak reproductive years (late 20s to early 30s).^1^ Delaying childbearing can have significant consequences, including higher rates of infertility and age-related pregnancy complications.^2,3^

The literature exploring the decision to delay childbearing during a medical career is limited. Difficulties associated with pregnancy and childrearing during training include intense workload, staffing shortages, time commitment, health concerns, high stress levels, poor lactation support, limited mentorship, and limited financial compensation.^4,5^ One contributory aspect is the change in modern family structure and matriculation of females into medical training; traditional work hours (60 to 90 hours) and sacrifice expected within a medical career may no longer be compatible with the current workforce and personal values.^6,7^ This has led to increasing tension for those wishing to family build and parent during training.^7^

Despite adequate financial parental leave support for residents in Ontario, Canada (which is a major barrier described in other countries), a large population-based study demonstrated that physicians in Ontario delay reproduction during training.^1^ The aim of this study was to understand what factors play a role in the decision on when or whether to family build (ie, family planning) among residents or fellows. With a broad goal of understanding how to guide further advocacy and systemic change, we qualitatively characterized the experiences of trainees across various specialties and explored their thoughts regarding family building, access to family planning supports, workplace culture, and attitudes toward family planning.

## Methods

### Study Design

This qualitative study followed the Consolidated Criteria for Reporting Qualitative Research (COREQ) reporting guideline.^8^ This study was approved by the research ethics board at the University of Toronto. Informed consent for publication was obtained prior to the start of the interview. We performed one-on-one virtual interviews to obtain in-depth accounts of participants’ experiences, and performed thematic analysis.

### Setting, Sampling, and Recruitment

Network sampling was used via an email sent to 6 deans of the postgraduate medical education programs across Ontario, Canada, which was then distributed to postgraduate learners. Interested participants were asked to contact the study team and complete a demographic surgery that included age, sex, sexual orientation, specialty, year of training, and institution. Purposive sampling of 29 participants from 80 eligible trainees was used to ensure diverse representation with a priority on specialty and sex. Final sample size was based on thematic saturation, achieved after 25 interviews; no further themes emerged after 4 additional interviews. Participants received a $25 gift certificate.

### Data Collection

The semi-structured interview guide was informed by a detailed literature review (eAppendixes 1 and 2 in Supplement 1). The guide was adjusted by the research team after the initial 3 interviews, which were conducted by a reproductive endocrinology and infertility fellow (S.D.) or second year medical student (A.K.) via video conferencing between May to August 2022. S.D. and A.K. are female learners who had children at different stages of training. The risk of personal experiences biasing the results was reduced through bracketing and review of the analysis by the entire study team. Verbal consent was obtained. We assessed family planning or building goals, workplace culture, and knowledge of residency program supports for family planning. Interviews lasted 30 to 90 minutes, and interviews were transcribed verbatim by a transcript writing service using video conferencing recordings. Transcripts were checked for accuracy.

### Data Analysis

Transcripts were analyzed and coded using NVivo version 12 (Lumivero) software independently by S.D. and A.K. All authors have previous experience conducting and publishing qualitative research. Specifically, D.G. has more than 15 years of experience in qualitative research, including design, data collection, analysis, and dissemination of findings. We conducted thematic analysis using open coding, constant comparison, and axial coding to identify common and divergent themes. We characterized the entire dataset according to major categories, such as perceptions and discussions around family planning and available supports in training.^9,10^ Coding strategies and themes were discussed and compared in depth by S.D. and A.K., with input from the full research team, to ensure rigor of the methods and accuracy of the results.

## Results

There were 29 participants (median [range] age, 30 [25-37] years), 22 females and 7 males. The participants included both 24 residents (82.8%) and 5 fellows (17.2%) and 8 (27.6%) were from surgical specialties, 3 (10.3%) from pediatrics and subspecialties, 4 (13.8%) from internal medicine and subspecialties, 2 (6.9%) from obstetrics and gynecology, 3 (10.3%) family medicine, 2 (6.9%) from anesthesia, and 7 (24.1%) other medical specialties. Broad representation by specialty and family planning goals was achieved (Table 1). The overarching theme was that the medical culture has not adapted to support medical learners in family building, leading to personal and professional dissonance. Four supporting themes were identified: (1) tension between role as a physician and role as a parent; (2) impact of role models and mentorship on family planning choices; (3) family building is discouraged during training, especially in surgical specialties; and (4) strong desire for tangible family planning supports in training.

**Table 1.** Demographic Characteristics of the Study Sample of Resident and Fellows (N = 29)

Characteristics	No. (%) (N = 29)
Sex	
Male	7 (24.1)
Female	22 (75.9)
Sexuality	
Heterosexual	26 (89.7)
LGBTQ+	3 (10.3)
Age, median (range)	30 (25-37)
Specialty^a^	
Surgery^b^	8 (27.6)
Pediatrics and subspecialities^c^	3 (10.3)
Internal medicine and subspecialties^d^	4 (13.7)
Obstetrics and gynecology	2 (6.9)
Family medicine	3 (10.3)
Anesthesia	2 (6.9)
Other medical specialties^e^	7 (24.1)
Residency or fellowship training institution^a^	
University of Toronto	17 (58.6)
Queens	8 (27.6)
NOSM	2 (6.9)
Ottawa	2 (6.9)
Level of training^a^	
Residents	24 (82.8)
Fellows	5 (17.2)
Parental status^a^	
Have child/ren	12 (41.4)
Intend to have child/ren	11 (37.9)
Deferred decision	6 (20.7)

Abbreviations: LGBTQ+, lesbian, gay, bisexual, transgender, queer, and questioning; NOSM, Northern Ontario School of Medicine.

^a^ Percentages may not add to 100 because of rounding.

^b^ Urology, neurosurgery, ophthalmology, general surgery, and orthopedics.

^c^ Infectious diseases.

^d^ Rheumatology, nephrology, and medical oncology.

^e^ Dermatology, radiology, emergency medicine, neurology, physical medicine and rehabilitation, and psychiatry.

### Theme 1: Tension Between Role as a Physician and Role as a Parent

Participants emphasized the rigor and dedication required to become a physician and highlighted that the expectation of this commitment to the profession seemed different compared with other careers. Participants described that success and reputation rely heavily upon their career dedication and personal sacrifice, particularly in surgical specialties. This culture of demonstration of dedication has led many trainees to feel that becoming a parent means assuming a different role that would pull them away from their primary role as a physician. Trainees with children shared that leaving work for family obligations was viewed negatively by peers and supervisors.

Trainees expressed that they feared family building in training would affect performance in either their role as physician or parent. They feared that they will be seen as distracted at work, and they will miss out on key moments with their children at home. The underlying sentiment was that no matter how competent a trainee may be, they are less likely to be taken seriously once they have children. The need for career prioritization was apparent in the way some trainees approached family planning and building. Trainees spoke about the need to build a reputation as a good resident or fellow before asking for any leniency. Many described a strong desire for control and developed a family plan that perfectly aligned with their training trajectory. Some expressed disappointment that their timelines were not achieved, and they experienced infertility and pregnancy loss. Others simply avoided family planning, feeling too overwhelmed by the notion of starting a family during training and subsequently described decisional regret when they reached the end of their training.

Resentment for the perceived family building constraints was expressed by several participants. Participants felt that the power to choose when to start their family was taken away from them. This was emphasized by those in surgical specialties due to longer work-hour and physical demands. Some expressed jealousy toward those in nonsurgical specialties or in nonmedical professions as it appeared they were able to meet life milestones sooner. One participant spoke about egg freezing as a demonstration of this unsupportive culture as it perpetuates the idea that family building should only be done when it is convenient within one’s career.

Participants who had children during training felt they had to overcome barriers. They described strong personal motivation to start their families in training, often describing that they entered medicine with the concept that they would have children and balance was possible, referencing childhood experiences of growing up with a physician parent or a family-oriented personal culture. Table 2 includes examples of quotes to illustrate theme 1 further.

**Table 2.** Tension Between Role as a Physician and Role as a Parent

Subtheme	Supporting quote
Having a medical career and a family are at odds with each other	“If you’re focused on a career like medicine, it shouldn’t hinder you from having the ability to have kids. And I think people really think of that as one or the other and I don’t think that that should be the case.” Participant 036, female, obstetrics and gynecology
	“But when is the right time and does the right time mean, you’re pushing yourself into a time where it becomes harder to have a child? I think it’s challenging because I am interested in having a family and don’t want to push it off to too far to where becomes increase increasingly challenging.” Participant 013, male, surgery
Reputational harm	“So, one of my colleagues from another [surgical] specialty, she actually had three children during her residency. She was one of the best residents I’ve ever worked with. And I remember one of the staff in her program making a joke about her, about how many children she’s had during the entire residency program. And I was just, at the time a medical student kind of hearing this, and I was like, this is kind of not OK.” Participant 017, female, surgery
	“I collected a series of additional evaluations to prove that objectively, so that if anyone says you’re not performing at the level you should be, I’d say here are 50 evaluations from various surgeons that say otherwise. And I felt that I had to do that to kind of almost, in a way, prove myself, so that people wouldn’t contest that I had taken time off.” Participant 033, female, surgery
Effect of career on family planning	“My program is a four year program, and we had thought, we’ll plan it so we have kids early in third year. This was our thinking, we could meticulously plan every detail of how this would work out. And then, when we started actually trying, it was just month after month unsuccessful.” Participant 011, female, pediatrics
	“I feel as if I’ve put the vast majority of my adult life towards the training to become a physician and I’ve had to put on the back burner my own personal goals in terms of getting married and having kids and it’s entirely because of medicine and the constraints that medical training puts on your life. That’s completely altered my trajectory in terms of what I could have done or might have wanted to do, so as a result I’m not married and I don’t have a child, when had I not been in medical training, I probably would have done all those things already.” Participant 037, female, internal medicine subspecialty
	“I don’t support the freezing of eggs, but just the idea that like, I can decide when I’m going to have a kid, and I have control over that. It makes the whole process of like having a family and like children, a product to consume. You have no real control over any of this. I don’t like selling the idea that you have control.” Participant 019, female, family medicine
Feeling overwhelmed	“I don’t necessarily have good suggestions on how to make it easier for women to actually have babies in residency just because I find that concept so overwhelming. It’s not the time around having the baby that’s so challenging, it would really be like the day to day of residency needs to be better. I always said to myself, I cannot care for another human being if I cannot even care for myself right now. So there’s just so much to tackle at the general residency level to make residents quality of life better.” Participant 032, female, surgery
Work culture	“It was a younger male and there was a comment about ‘we’re now used to the chaos with so many people taking maternity leave’ or something. Not a really negative comment, but not really a positive comment. And it stuck with me, because it felt like, so you consider maternity leaves chaos. So, just small remarks here and there that were not viewed as a big deal can actually be not so welcoming. But again, that was just one incident, but it really did stand out, I haven’t forgotten it.” Participant 017, female, surgery
	“It’s really part of our culture to just always be there, always stay post-call, like never leave, and I can’t do that, I have to see my family. And then I was pregnant again and I was like I can’t stay post-call. I’m like 500 pounds, I can’t move, I had some medical complications, and so I need to get home. And that doesn’t look good, too, right? Because you can’t compete with people that are there all the time.” Participant 033, female, surgery
	“I was on my phone trying to figure out childcare, like who’s going to pick up my child because I was late. And then my preceptor thought it was unprofessional. She didn’t know what I was doing. She wrote it in my evaluation. I told her later what the case was because she had brought it and she was like, ‘Oh, I wish you would have told me sooner.’ I didn’t get a chance. But if she hadn’t brought it up that she thought it was unprofessional, then I wouldn’t have been able to say anything. It’d be nice to be given opportunities to bring it up.” Participant 019, female, family medicine

### Theme 2: Impact of Role Models and Mentorship on Family Planning Choices

#### Mentorship Shapes Perceptions

Participants had varying mentorship experiences. Some felt uncomfortable discussing family planning with supervisors. Articulated reasons included concerns about crossing personal lines, fears about judgement or being viewed differently, and concerns about the effects on future opportunities, including hiring potential. Participants who found mentors who directly discussed family planning or actively modeled work-life balance were more likely to feel that family building during training was possible. Conversely, those who had mentors who expressed negativity, even just in passing, discouraged trainees from engaging in family planning.

The participants discussed 2 ways that having a mentor who was open about family planning played a role their career and family decisions. First, they provided hope that balancing family and a medical career within that specialty was possible. In some cases, this factored into a trainee’s decision to pursue that specialty. Second, these mentors often provided very specific and applicable advice needed to make their own family planning and career decisions. In particular, mentors of the same sex who could role model behaviors were identified to be the most influential.

#### Limited Male Mentorship

Although they expressed the desire to have these discussions, male participants felt their mentors were less likely to discuss family planning topics with them. If conversations around family planning did occur, they were more often initiated by the male trainee. They attributed this to a lower biological time pressure to have children and speculated that mentors may have assumed that these conversations were less applicable to male trainees. Direct quotes that emphasize key points from theme 2 can be found in Table 3.

**Table 3.** Impact of Role Models and Mentorship on Family Planning Choices

Subtheme	Supporting quote
Importance of role models	“One of my mentors did describe the challenges of being pulled to work late hours and long days regularly vs the pressures of family life and trying to maintain that balance. And it was actually quite inspiring the hear him describing some of the ways he was able to manage that and what he prioritized and making professional sacrifices in order to do that. The ultimate message being that he never once regretted the time he spent with family. So that, to me, was a real role model from that perspective, as well.” Participant 013, male, surgery
	“And then, looking back on it now, I had Dr. X, who’s an orthopedic surgeon telling me about how she had kids while doing her orthopedic training, and how that was all possible, and how she had 2 daughters and her husband was also in medicine. And it all seemed very feasible and very doable at the time. Of course, now in 2020, she’s the only woman we have who has children in our entire division. So maybe a little naive on that front when I assessed that, but that’s how I ended up in ortho.” Participant 030, female, surgery
Positive role models/mentorship demonstrate family building is possible	“I have a few coresidents actually, who do have children during residency. To me it seems quite challenging. It’s a very busy residency and to be able to dedicate that time to family, or doing other things, while dealing with call and all the studying and the work and the surgical training, it just seems very challenging. And I’m sure is very doable, because I’ve seen a lot of them thrive in residency and in their family life but yes, if the timing was right and I had, I guess—or if I wanted to start a family—I don’t think I would consider residency a barrier to do so.” Participant 089, female, surgery
	“I knew that the specialty that I’m going into is supportive for family and family planning. I came to that conclusion as I did my clerkship in the specialty so that many of the residents who were going through residency actually started family or in the process of starting families. And that’s different from what I observed in different specialties.” Participant 16, male, pediatrics
	“I have a female program director who had multiple children in residency, and there are a lot of other women female emergency doctors who have had kids within the past 4 years. So, I set up meetings with them, and they were very happy to take me for a coffee, take me for a meal, and discuss their experience with that. So, there are a ton of people who have been excellent mentors along the entire process, starting from me making the decision to have a child, all through the pregnancy, and now as I’m discussing return to work, they’ve been with me all throughout that process.” Participant 060, female, other medical specialty
Lack of mentorship is challenging	“I think it’s probably too forward, one I wouldn’t feel comfortable talking to any of my staff about wanting to have children because I just don’t feel that they would really understand where I’m coming from. And then in terms of female staff, I still don’t feel comfortable speaking to them. I think if you don’t know someone well enough, I don’t really want to intrude.” Participant 090, female, internal medicine
Female-specific mentorship	“I think it’s probably more on the top of people’s minds for a female, so I think that my experience was being a male, nobody has really brought that to me as an issue.” Participant 013, male, surgery
Negative comments are influential	“I was advised by a few people that it would be hard to have a family and things like that if I did surgery....And I think sometimes it also comes from the fact that these people might have gone through some hardships in their personal life.... Going all the way back to just choosing to do medicine, I was at work and I found out I got into med school. One of the lab techs that I was working with as a summer research student said, ‘Oh well you won’t be able to have children now.’ And that kind of deflated me.” Participant 023, female, surgery

### Theme 3: Family Building Is Discouraged During Training, Especially in Surgical Specialties

#### Trainees Perceive Lack of Formal Dialogue About Family Planning as Unsupportive

Overall, participants perceived that family building was discouraged during training. Participants shared that while there were optional learning events, no respondent had formal teaching on the topic in medical schools or residency.

Without formal discussion and availability of resources, trainees described feeling responsible to sift through sources of information and piece together what is available to them during an already busy and stressful time. Participants stated that not publicizing these supports gave the impression that family building was undesirable, even though programs would assist trainees if needed.

Participants identified their program directors as being important representatives of program culture. Participants who felt poorly supported discussed that they had hoped that their family planning goals were brought up by their program director.

#### Family Building Negatively Impacts Other Trainees

Some participants were comfortable talking to colleagues and felt that being in the same position made them most understanding of the concerns. Others were worried that they’d be seen as less committed, or that their colleagues would resent them having a child as it would increase others’ workload. Many trainees expressed guilt around extra responsibility placed on peers because of their pregnancy or leave, particularly among those in smaller programs. Learners feared leaving their cohort as they perceived that if they were off cycle, they would receive less academic supports from their program. This was shared in the context that programs often have a rigid structure that requires a cohort to move through rotations on a set schedule.

#### Supportive vs Unsupportive Programs

Some participants who chose to have children during training or engaged in fertility preservation or treatment did feel supported by their program. They perceived this support through the sharing of birth announcements, regular reminders of available resources, and open conversations about children between faculty and trainees. This differed by program and experience with prior trainees in the same position. Those programs in which a learner was 1 of the few engaging in family building felt that the onus was on them to create a support system and tell the program what was needed; they resented this position. There was less perceived support in surgical specialties. Some areas of lack of perceived support included the lack of the ability to arrange time for medical appointments, lack of graduated return-to-work, requirement to be on-call during later stages in pregnancy, and the perception of a parent being less dedicated to the profession. Direct quotes relating to theme 3 can be found in Table 4.

**Table 4.** Family Building Is Discouraged During Training, Especially in Surgical Specialties

Subtheme	Supporting quote
Formal family planning discussions demonstrate support	“I think it [formal discussions] would definitely help think more about their own personal life and their relationships in the broadest sense so that they can know it’s not only like tolerable for them to be thinking about these things but in fact it’s favorable, I think.” Participant 057, female, other medical specialty
	“It’s not systemically in place. Every 6 months we have a review with our program director and I don’t believe once that’s ever come up as an issue. And I’ve given her the feedback, I’m like, ‘Maybe you should ask about how are your relationships?’ you know, a little bit more of a relational perspective in the broader sense of what relationality means.” Participant 057, female, other medical specialty
Hidden curriculum	“I think generally in medicine there is a—what’s it called—like an attitude that family planning is kind of swept under the rug and you should keep it secret until you have your position and never mention it on the interview trail and kind of keep it under wraps and there is a lot of secrecy around it.” Participant 037, female, internal medicine subspecialty
	“I guess it’s reassuring that everyone’s kind of thinking along the same lines. It’s demoralizing that we all feel this way. Surgery in general does not feel like a supportive community when I hear so many females of my age range. And it’s nothing specific about my program. It’s just that it’s really hard to be a surgical resident or any resident, but just from what I know, it’s really hard being a surgical resident.” Participant 032, female, surgery
	“And it’s hard to pin down and I don’t think anyone would ever say it out loud because it doesn’t manifest itself obviously. It doesn’t manifest itself in people talking about it explicitly, it manifests itself in subtle cultural changes in the way people treat you and what things you’re invited to and what things people talk about with you and that’s what I’ve noticed.” Participant 001, female, other medical specialty
	“Yeah, I feel like there needs to be a bit more like, almost like normalizing of the culture that doctors are people who have families and kids, and many of them are women. Classically we think of that traditional kind of viewpoint of medicine as being the old white man and so they’re always working and like they come home to their wife, and their wife isn’t the doctor but of course like, there’s like, there are many women in medicine now and the thought that they also will have kids, like that should be normalized right.” Participant 073, male, surgery
Fear of losing learning support if no longer with cohort	“And I think that with the program, if you don’t fall securely in that box of, you’re this cohort of residents, and you have to bend the schedule around you, I feel maybe you’re just not watched or regarded quite as closely.” Participant 086, male, pediatrics
Guilt of burdening co-residents in surgical specialties	“And when I was looking more at surgical specialties, there were some programs, especially smaller programs, that, you know, maybe it wasn’t staff, but at least the residents—somebody sort of explicitly said, it’s very hard, it’s very hard to get pregnant, to take maternity leave in this program, because you feel like you’re letting down the team because it’s a very small residency program, and people are going to have to cover for you and pick up the slack.” Participant 061, female, other medical specialty
Expressed burden to colleagues in other specialties	“I think there is a stigma to it and there are some people that are of the opinion that it’s unfair for people to have children during residency or early part of their career or in the academic studying because it puts extra burden on your colleagues.” Participant 037, female, internal medicine subspecialty
Acquiring knowledge about family planning supports is the responsibility of the trainee	“I don’t think the program really has brought anything forward, I think it’s all been through kind of self-investigation into documents or policies or things that are available online. If that [family planning resources] could be presented and know where kind of those resources are, instead of having to try to dig them up or find them, that would be useful.” Participant 055, male, other medical specialty
Lack of support upon return from parental leave	“And I felt that I was very, very well supported when I was pregnant, from my program, but I felt that when I had a child and I came back, there was just no mechanism in place to deal with that.” Participant 033, female, surgery
	“So it’s like—well really what, ‘Is it really worth it to come back to residency? Why don’t I just stay home and care for my child.’ Because your kid is not going anywhere, they still need a lot of attention and care. And yet add on to that the rigorous of residency, and it’s just really, really hard to balance everything.” Participant 66, male, medicine

### Theme 4: Need for Tangible Family Planning Supports in Training

#### Role Models Are Valued

Participants emphasized the importance of tangible supports for family building during training to empower learners in creating appropriate family plans. Trainees in programs where there had been role models ahead of them felt more comfortable engaging in family building because there was more of a positive perception that someone had done it before. Trainees noted that they looked at programs more favorably during the selection process when trainees who had children during training were highlighted as a demonstration of the respect of wellness.

#### Financial Barriers to Family Building

Fears expressed by trainees included a loss of skill and knowledge during parental leave with possible supports including a graduated return to work. Financial support was a big concern as the costs of raising a child on a trainee’s salary, income loss during leave, and high debt and repayment needs were all described as barriers. Some participants had partners who were also trainees; they specifically commented on difficulties coordinating schedules and childcare, financial burdens and an inequitable parental role that positioned the female partner as a primary parent (ie, different leave lengths, different schedule flexibility).

#### Desired Supports

Trainees expressed a desire for more support in attending medical appointments during fertility treatment and pregnancy that did not require the use of a vacation day. In addition, they expressed the desire for more financial support for engaging in fertility treatment or preservation. Participants highlighted the need for schedule accommodation depending on trainee preference and pregnancy stage, protection from potentially harmful exposures, protected study time for required examinations, and extra compensation for peers covering redistributed call to alleviate resentment. Trainees specifically discussed the lack of support available upon return from parental leave. Participants stated that equitable parental leave, accommodation of working hours to affordable childcare options (ie, daycare hours), on-site childcare, mentorship when returning from leave, lactation resources when returning to work, and options for part-time residency were all supports that would make family building in training more accessible. Direct quotes relating to theme 4 are in Table 5.

**Table 5.** Need for Tangible Supports Within Training Programs

Subtheme	Supporting quote
Actions speak louder than words	“I think it depends on how you’re defining support. If you mean in the sense that they’ll be happy for you and they’ll find people to cover your shifts and let you take a maternity leave, yeah but I don’t know that it goes that much further than that. As far as what tangibly it looks like for making your life easier when you have children, I don’t know that that’s happening.” Participant 001, female, other medical specialty
Need for protected time off, without subjective evaluation of validity	“I couldn’t even get time off, like it was brutal trying to even just organize it [egg freezing]...I took a week off as one of my holidays, like one of my four weeks off, I took it off to do this. And I was like, this isn’t a freaking holiday. I shouldn’t have to use my protected vacation time to do this.” Participant 032, female, surgery
Transparency and flexibility around the scheduling to accommodate family building and parenting	“If I were pregnant, I wish that earlier on the rotations that are—like let’s say I’m on core OB then this is more demanding, I’d rather do it at the beginning than do it when I’m heavily pregnant, and kind of like organizing my rotations around that.” Participant 081, female, obstetrics and gynecology
	“I think a more transparent pathway of what residency might look like if you were to get pregnant in the different years, I think would be helpful. Knowing what it would look like if you took, for example, three months off versus a year off, that kind of—those options I think would be helpful.” Participant 047, female, anesthesia
	“But even just some better appreciation for the typical daycare hours or nanny hours. And how that is just impossible with a young child. Patient handover at eight and five just doesn’t work for most daycares and most nannies. Even like 4:30 handover to extend that night shift a little bit longer in order to get people out at five o’clockI think would help so many people with young children.” Participant 011, female, pediatrics
Fair compensation for extra call to limit resentment toward trainees for taking leave	“I think it’s very complicated because I think a lot of them to be fair, like feel frustrated and upset by the extra call that all of a sudden you have to do. And there’s always like almost zero compensation for doing this extra call that you wouldn’t have had to do otherwise. But I think blaming, putting that anger and frustration on the person taking the leave is not the right place for it to go to. And honestly, I think like fair compensation for having to take on extra call would change that feeling a lot amongst residents.” Participant 011, female, pediatrics
Support around return to work and graduated responsibility	“Because you know, it’s been over a year since I’ve done them now and I’m feeling a little bit nervous about delving back into it. So, it’s kind of a remote place to be in because I’m in final year and I ended my PGY-5 year, a year ago I was locally, independently and felt very confident and most of that confidence is gone now.” Participant 090, female, internal medicine

## Discussion

Overall, the medical culture is not perceived as supportive for individuals who are family building, which leads to tension between roles as a physician and as a parent. This culture persists from medical school to postgraduate training.^11,12^ Prior work shows that medical trainees have poor knowledge of age-related fertility decline and the risks of advanced age in pregnancy.^13,14,15^ Our study identifies that trainees feel unsupported in actively engaging in family building due to the lack of explicit information and fear of harming their reputation. Many trainees viewed fertility and family planning as within their control, leading to disappointment when they discovered that it would not fit within the strict timelines of their training. Studies show that more than half of physicians regret delayed childbearing.^16^ Trainees who receive education on the risks of infertility and delayed pregnancy are more likely to have pregnancies earlier in their career and less likely to require fertility evaluations or treatment.^17^ This highlights the need for transparent information regarding age-related fertility decline for all physicians and available resources beginning early in their career to assist trainees in making family planning decisions.

Our study highlights the tension that physicians face between being a physician and parent. Parenthood is seen as an inconvenience to a medical career.^16^ Participants who did pursue family building during training often had positive role models, however, they still felt that their decision represented one that pulled them away from the dedication required for a successful career. Importantly, this was not based on appreciable skill decline but rather their colleagues’ perceptions. Learners who did not have role models or prioritized their career over having a family struggled to identify how they could balance their goals of parenthood with their career and felt uncomfortable asking other learners or attending physicians in their program as this seemed too personal. Our study echoes previous research identifying that role models and mentors are important for medical trainees considering family building; participants who connected with mentors or role models had a positive perception of family building within training.^17^ Participants who identified mentors prior to residency selection considered this in their specialty selection; participants who saw learners within the program that had children felt like this was possible for them as well. Importantly, our study adds that this is important for all sexes, as males have traditionally been excluded from these discussions. There are also more family structures with both parents as physicians, highlighting the need for discussions around equitable home contributions and professional development for all genders.

Lastly, participants emphasized the need for tangible family planning or building supports. Despite a generous paid parental leave policy (ie, employment insurance up to 18 months), participants still felt that family building was not supported. This highlights that paid parental leave is not enough to create a supportive culture for trainees to consider family building. In addition, despite reasonable salaries for residents in Ontario, there are high costs of living, high costs for childcare (especially at extended hours), and high debt repayment. Many learners feared overburdening their colleagues and struggled to understand how they could handle their current workload if they had a child. Suggestions for adequate future workforce planning include a critical financial analysis of duty hours and the concordance to the provision of adequate childcare and personal fulfilment. Our study echoes other studies which call for tangible supports including adequate clinical human resources, incentivization of call coverage, appropriate work hours for childcare, and flexibility in rotation scheduling.^5^

### Limitations

This study has limitations. It is possible that not all viewpoints were captured and other career stages were not sampled; participants who had different opinions may not have pursued the opportunity to participate. This study was limited to 1 province in Canada, which may not be applicable to other jurisdictions. Next steps include implementing and assessing specific interventions to improve family planning support for trainees.

## Conclusions

The medical culture is not perceived to be supportive for trainees who engaged in family planning, family building, and parenting. This leads to individual tension as they struggle to find a place for their roles outside of medicine within their medical career. Although there are protective rules and policies for parental leave, there remains a general discouragement inherent in the medical culture. Opportunities to foster a supportive culture include providing transparent information on family building in medicine, fostering mentorship, and ensuring appropriate workforce planning, with individuals who have caregiving roles taken into consideration.

## Notes

Supplement 1. **eAppendix 1.** Demographic Survey **eAppendix 2.** Interview Questions

Supplement 2.
Data Sharing Statement
