# Supplementary material for: Resident and Fellow Perspectives on Family Planning and Building During Training
Source: JAMA Netw Open. 2024 Aug 28;7(8):e2429345. doi: 10.1001/jamanetworkopen.2024.29345 (PMC11358854; doi:10.1001/jamanetworkopen.2024.29345)
Supplement: Supplement 1. — eAppendix 1. Demographic Survey eAppendix 2. Interview Questions [file jamanetwopen-e2429345-s001.pdf]

Supplemental Online Content

Dason ES, Kapsack A, Baxter N, Gesink D, Shapiro H, Simpson A. Resident and fellow perspectives on family planning and building in training. *JAMA Netw Open*. 7(8):e2429345. doi:10.1001/jamanetworkopen.2024.29345

eAppendix 1. Demographic Survey

eAppendix 2. Interview Questions

This supplemental material has been provided by the authors to give readers additional information about their work.

© 2024 [First author last name and initials or group name] et al. *[Journal name].*

eAppendix 1: Demographic Survey

1. What is your name?
2. What is your email address?
3. What is your age?
4. What is your gender?
5. What is your sexual orientation?
6. What is your specialty?

eAppendix 2: Interview Questions

The following questions explore your personal thoughts on family planning and your knowledge on fertility changes and treatments. Please consider the answers as they pertain to you, not to future patients you may be counselling. You may choose not to answer questions or take a break if you need to. It may be difficult to share your experiences with me.

1. What were some of the non-professional factors that contributed to your residency program and specialty selection (e.g. family planning, lifestyle, work-life balance)
2. How did considerations around the hours worked or length of residency play into your decision to select a specialty/program?
    a. Which of these factors (ie. Family planning, lifestyle, work-life balance, hours worked, length of residency) would you say was the most important?
3. Did you consider whether or not your specialty would have family planning supports in place when selecting your residency?
4. How did you seek information about family planning within specialty you applied to?
    a. i.e. information provided by the program, conversations w current residents
5. How did you seek information about family planning in the residency programs you applied to?
6. Did you have any specific mentors that you sought information from?
    a. What made you connect with your various mentors?
7. What factors did you consider most important when you were ranking programs?
    a. Did you consider the "culture" of the program?
    b. ***How did you develop your impression about how supportive your speciality was regarding family planning?***
8. How did your relationship status impact your residency specialty or program decision?
9. How has your career path thus far impacted your family planning?
10. What is your impression about having a child in residency?
    a. **what has your experience been with having a child in residency?*
    b. *What supports were available to you when you were returning to work*
    c. *What supports would you have liked to have seen?*

© 2024 [First author last name and initials or group name] et al. *[Journal name].*

    d. Did you consider being pregnant/fear of complications, needing time off for appointments, finding coverage or losing skills during leave, being a working parent?

11. What is your impression about having a child as staff?
    a. Did you consider being pregnant/fear of complications, needing time off for appointments, finding coverage or losing skills during leave, being a working parent?
12. Do you have any mentors who you have spoken to about family planning during residency?
    a. How did you find these/this mentor?
13. Is your residency program supportive of residents having children during residency ?
    a. What supports are available within your program to residents having children?
    b. What supports are available to residents within your program who want to preserve their fertility?
    c. Do supports available within your program differ by gender?
    d. How has your program shared details of this support with you ? (e.g. explicit/implicit, when during training)
    e. What kind of supports would you like to see in your residency program in regard to family planning?
14. Would you feel comfortable discussing family planning with your co-residents?
15. What supports are available at the University (outside your program) to residents having children?
16. Do you know what supports are available at the University (outside your program) to residents wishing to preserve their fertility?
17. Do you know what supports are available at the provincial level (i.e. PARO) to residents having children?
18. What supports are available at the provincial level (i.e. PARO) to residents wishing to preserve their fertility?
19. Would you feel comfortable discussing family planning with the staff and faculty at your residency program?

Fertility-related Questions

1. What do you know about family planning and fertility?
    a. How would you rate your knowledge on family planning and fertility, 1 to 10?
2. What is your understanding of age-related fertility changes?
    a. What are your thoughts about age-related fertility changes in the context of your own life? When do you think your fertility will decline?
3. Was having children in the context of a career in medicine discussed formally during your training? (ie. Lectures, material)
    a. Was it ever informally discussed? Preceptors, co-residents, family?
    b. What was the attitude of those with who you discussed this?
    c. Did you seek out information/mentorship around these issues during residency? Before residency?

d. What did you learn and how did what you learned impact your perceptions?

4. Would you tell me your what your thoughts are on when you might choose to have a family?
   a. What factors influenced your decision regarding when to have a child?
      i. Did your age, gender, sexuality, ethnicity or religious beliefs have any impact on your goals?
   b. What is your understanding of the family building options that are available to you? (i.e. spontaneous conception, IVF, egg-freezing, adoption, surrogacy)
   c. Have you considered whether you might personally encounter fertility issues?
   d. *Have you considered fertility preservation (i.e. egg freezing or IVF) for you or your partner during training?*
      i. Is there an age at which you would you think eggs should be frozen if you are not ready to have children?
      ii. What would motivate you or your partner to freeze your eggs? What would stop you or your partner from freezing your eggs?

Ending :

In an ideal world, how could the medical system support trainees engaging in family planning at all stages?

Are there any other thoughts or comments that you'd like share with us regarding your experiences or your suggestions for family planning supports going forward

Do you have any questions for us?

Thank you for the time you've taken to share your thoughts with us.

© 2024 [First author last name and initials or group name] et al. *[Journal name].*
